# Cellular attachment and osteoblast differentiation of mesenchymal stem cells on natural cuttlefish bone

**DOI:** 10.1002/jbm.a.34113

**Published:** 2012-07

**Authors:** Beom-Su Kim, Jin Seong Kim, Hark-Mo Sung, Hyung-Keun You, Jun Lee

**Affiliations:** 1 Wonkwang Bone Regeneration Research Institute, Wonkwang UniversityIksan 570-749, Korea; 2 Department of Periodontology, School of Dentistry, Wonkwang UniversityIksan 570-749, Korea

**Keywords:** cuttlefish bone, mesenchymal stem cell, biocompatibility, scaffold, bone tissue engineering

## Abstract

The purpose of this study was to describe an approach that aims to provide fundamental information for the application of natural cuttlefish bone. Before applying cuttlefish bone as a bone defect filling material, we evaluated proliferation, adhesion, and cell viability of human mesenchymal stem cells (hMSCs) cultured on cuttlefish bone. Cuttlefish bone was separated into two parts (dorsal shield and lamellar region) and each part was used. Cell proliferation and viability were assessed using the MTS assay and live/dead fluorescence staining method. The morphology was observed using scanning electron microscopy (SEM). hMSCs were stimulated with osteogenic medium and osteoblast differentiation was evaluated. The fluorescence images showed that the seeded cells grew well and that cell distribution was in accordance with the surface morphology of the cuttlefish bone. Compared with the dorsal shield, cells penetrated deeper into the three-dimensional inner space of the lamellar part. Furthermore, under osteogenic differentiation conditions, alkaline phosphatase activity increased and the mRNA expression of ALP, runt-related transcription factor 2, and collagen type I α1 was increased in hMSCs cultured on both the dorsal shield and lamellar block. These results indicate the potential of cuttlefish bone as an ideal scaffold for bone regenerative materials. © 2012 Wiley Periodicals, Inc. J Biomed Mater Res Part A, 2012.

## INTRODUCTION

Numerous conditions, for example, regeneration of damaged bone resulting from disease, trauma, and infection, require bone graft replacement. Despite recent results obtained in bone regeneration and bone graft transplants, there are still crucial issues to be resolved. Although autologous bone grafts have been used as a gold standard for surgical procedures, this approach has some disadvantages, for example, the need for a second surgery at the donor site and limited amounts and shape of bone.^1^ To overcome these problems, tissue engineering has emerged as a promising approach to treat the lost or diseased bone. The most common approach of bone tissue engineering is based on the concept of developing new biomaterial scaffolds. The role of the scaffold is to provide cells an environment suitable for their proliferation and differentiation to induce tissue regeneration. Therefore, over the past decade, the main goal of bone tissue engineering has been to develop scaffold materials for substitution of autografts for filling large bone defects. In addition, this approach combines cells capable of osteogenic activity with a suitable scaffold material to regenerate bone.^2^ In particular, mesenchymal stem cells (MSCs) have a self-renewal capacity and multilineage potential. They can be differentiated into osteoblasts, and thus have been used to engineer bone tissue.^3^

In the bone tissue engineering area, there are several biomaterials that can be used as scaffolds, such as coral, fibrin, and demineralized bone.^4^–^6^ Among these, coral material is considered a particularly suitable biomaterial in bone tissue engineering due to its properties of osteoconduction, biodegradability, and biocompatibility.^4^ Cuttlefish bones have a similar chemical composition and crystallography to coral.^7^

Cuttlefish bone is the hard tissue in cuttlefish, which acts as a floating tank in the animal. Cuttlefish bone consists of two regions. One is a thick external wall (dorsal shield) and the other is an internal lamellar matrix. The dorsal shield is a thick, hard cover that overlays the lamellar matrix. The internal lamellar matrix consists of a parallel sheet structure of calcium carbonate and the spaces between the sheets (200–600 μm) result in highly porous properties.^8^ Cuttlefish bone is mostly composed of calcium carbonate. Therefore, several experimental studies have been conducted on natural cuttlefish bone as a calcium source for bone substitutes. General bone is composed of calcium phosphate, typically hydroxyapatite (Ca_10_[PO_4_]_6_[OH]_2_). Therefore, most reports have focused on the conversion of calcium carbonate materials to hydroxyapatite.^9^, ^10^ However, several studies have reported the use of calcium carbonate from natural aragonite as a potential bone substitute for the regeneration of bone defects.^11^, ^12^ These finding suggest that natural cuttlefish bone may also be used as a bone substitute without processing its calcium carbonate to hydroxyapatite. However, the use of natural cuttlefish bone may be hampered because of cytotoxicity and insufficient biocompatibility with human MSCs (hMSCs).

Therefore, the aim of this work was to demonstrate the biocompatibility and osteoblast differentiation of hMSCs cultured on natural cuttlefish bone. In this study, we used bone marrow-derived hMSCs, which we seeded and then subsequently evaluated for proliferation and attachment. In addition, cell osteoblast differentiation was observed using particular markers of osteoblastic differentiation during culture on cuttlefish bone.

## MATERIALS AND METHODS

### Preparation of cuttlefish bone blocks

Cuttlefish bone was removed from cuttlefish (*Sepia esculenta*, from the Korean western Sea), gently washed with distilled water, and dried. First, the dorsal shield and lamellar regions were separated from the cuttlefish bone. The samples were cut into blocks of 4 × 4 × 1 mm^3^ and 10 × 10 × 2 mm^3^. For cell culture experiments, these blocks were sterilized with ethylene oxide (EO) gas and transferred to tissue culture dishes.

### Mesenchymal stem cell culture

In this study, hMSCs derived from alveolar bone marrow were used to determine the behavior of cells cultured on cuttlefish bone blocks. To obtain hMSCs, bone marrow was aspirated from the alveolar bone of patients during oral surgery. All patients provided written informed consent and the study was approved by the Institutional Research Review Board of the Department of Periodontology at the Wonkwang University Dental Hospital. The general properties of hMSCs were confirmed in our previous study.^13^ Cells were cultured in α-MEM medium (Gibco-BRL, Gaithersburg, MD) containing 10% fetal bovine serum (FBS) and 1% antibiotics (penicillin G: 10,000 units/mL, amphotericin B: 25 μg/mL; Gibco-BRL) at 37°C and 5% CO_2_. In this study, cells of passages 3–6 were used and the culture medium was changed every 2 days.

### MTS assay

Proliferation of cells attached and grown on cuttlefish bone blocks was measured using CellTiter96® Aqueous One solution (Promega, Madison, WI). Briefly, hMSCs (1 × 10^3^ cells) were loaded on sterilized dorsal shield and lamellar cuttlefish bone blocks. After predetermined time intervals, 25 μL of MTS reagent was added to each well and incubated for 4 h. Absorbance was measured at 490 nm using an ELISA reader (SpectraMAX M3; Molecular Devices, Sunnyvale, CA).

### Viability and cytotoxicity assay

Cell viability and cytotoxicity were measured by staining using a Live/Dead® Viability/Cytotoxicity kit (Molecular Probes, UK). Briefly, hMSCs were seeded on cuttlefish bone blocks and cultured for 3 days. To remove phenol red and serum, cells cultured on cuttlefish bone blocks were washed with phosphate-buffered saline (PBS) for 30 min. The samples were then incubated in a Live/Dead® Viability/Cytotoxcity solution for 30 min. Calcein acetoxymethyl (Calcein AM, 0.05%)-stained healthy cells appeared green and ethidium homodimer-1 (EthD-1, 0.2%)-stained nuclei of dead cells appeared red. After staining, the samples were observed under a fluorescence microscope (DM IL LED FLUO; Leica, Wetzlar, Germany).

### Osteoblast induction

To induce osteoblast differentiation of hMSCs, cells were cultured in media supplemented with an osteogenic stimulant (OS), that is, 10 m*M* β-glycerophosphate (Sigma, St. Louis, MO), 50 μg/mL ascorbic acid (Sigma), and 0.1 m*M* dexamethasone (Sigma). The OS-containing media was changed every 2 days during osteogenic induction.

### SEM observation

To observe the morphology of cuttlefish bone and behavior of hMSCs on cuttlefish bone blocks, cells were seeded at 1 × 10^3^ cells/scaffold. SEM was performed on 3-day culture samples. The cuttlefish bone blocks seeded with hMSCs were washed with PBS buffer and fixed with 2.5% glutaraldehyde solution at 4°C for 16 h. The samples were postfixed with 0.1% osmium tetroxide solution and dehydrated in a series of ethanol (25%, 50%, 75%, 95%, 100%, and 100%). The dehydrated samples were sputter coated with platinum and viewed using SEM (JOM-6360; JEOL, Tokyo, Japan).

### Quantitative real-time-polymerase chain reaction (qRT-PCR)

To assess the gene expression of osteogenic markers, hMSCs were seeded at 1 × 10^5^ cells/scaffold. After 5 days of culture with OS-containing medium, total mRNA was isolated using a RNA purification kit (Ribospin™; GeneAll, Seoul, Korea) and cDNA was transcribed with reverse transcriptase (Invitrogen, Carlsbad, CA, USA) and oligo (dT) primers. cDNA was amplified using the TaqMan Universal PCR Master mix (Applied Biosystem) and primers and TaqMan probe sets for alkaline phosphatase (ALP; Hs01029144_m1), collagen type I α1 (ColIα1; Hs00164004_m1), runt-related transcription factor 2 (Runx2; Hs00231692_m1), and 18S (Hs99999901_s1) (Applied Biosciences). All TaqMan PCRs were performed using the StepOne Plus real-time PCR system (Applied Biosystems, Foster City, CA). The 18S rRNA gene was coamplified as an internal standard.

### ALP activity assay

The degree of osteoblastic differentiation of the cells was evaluated by determining ALP activity. After 5 days of culture using osteogenic induction medium, the adherent cells that were removed from the bone blocks were homogenized in PBS with 1% Triton X-100. A 100-μL aliquot of the suspension was then mixed with 200 μL of 0.1*M* glycine NaOH buffer (pH 10.4) and 100 μL of 15 m*M* *p*-nitrophenyl phosphate (*p*-NPP) (Sigma) and incubated for 30 min at 37°C. The reaction was terminated by adding 0.1*N* NaOH. The degree of *p*-NPP hydrolysis was determined using an ELISA reader (Spectra MAX M3) at 410 nm; *p*-nitrophenol (*p*-NP, Sigma) was used as a standard. Protein concentrations were measured using a BCA protein assay reagent kit (Pierce, Rockford, IL) according to the manufacturer's instruction. The experiment was repeated at least three times.

### Statistical analysis

All values were expressed as the mean ± standard deviation of the mean (SD). Statistical analysis was performed using Student's *t*-test and one-way ANOVA. *p* < 0.05 was considered to indicate statistical significance.

## RESULTS

**Figure 1:**
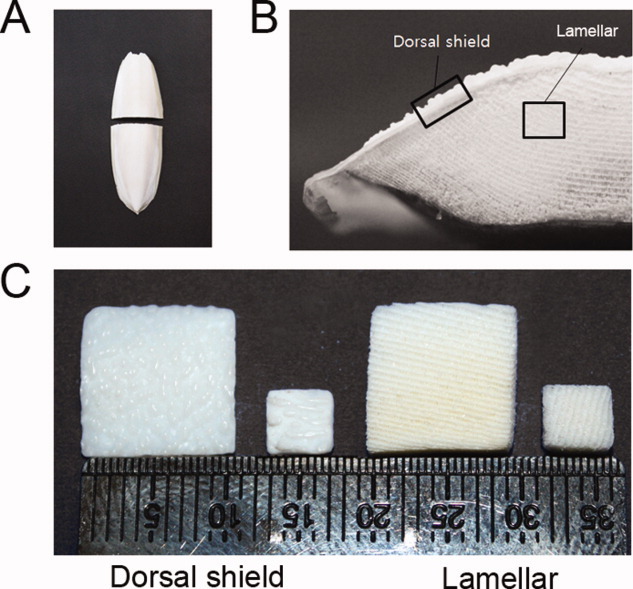
Preparation of dorsal shield and lamellar blocks from cuttlefish bone. A: Whole cuttlefish bone in planar view and transverse section. B: A digital image of the transverse section through the cuttlefish bone. C: Prepared cuttlefish bone blocks (10 × 10 × 2 mm^3^ and 4 × 4 × 1 mm^3^) for cell experiments. [Color figure can be viewed in the online issue, which is available at wileyonlinelibrary.com.]

**Figure 2:**
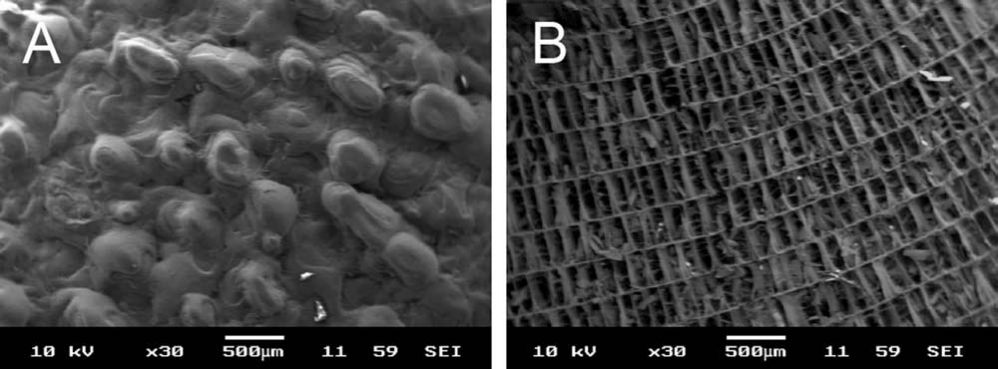
Scanning electron photographs of cuttlefish bone. A: Dorsal shield block. B: Lamellar framework of the transverse section of cuttlefish bone.

Cuttlefish bone was prepared and cut into small blocks (Fig. 1). To observe the structure of the lamellar and dorsal shield blocks, SEM was used. The results of SEM analysis showed that the dorsal shield blocks have an irregular surface and embossed nonporous morphology. In addition, numerous regular parallel sheets (∼500 μm) were observed on lamellar blocks (Fig. 2).

**Figure 3:**
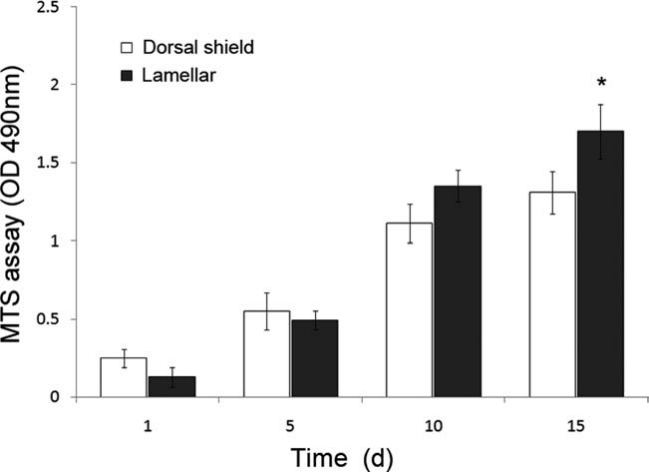
Cells were seeded on dorsal shield and lamellar blocks of cuttlefish bone and cultured in normal growth media. Proliferation profiles were evaluated using the MTS assay after 1, 5, 10, and 15 days of culture. Values are expressed as the mean ± SD. Significance was determined at *p < 0.05 when compared with dorsal shield blocks.

The representative graph for cell proliferation shows that both lamellar and dorsal shield blocks exhibit a similar cell growth pattern during cultivation. At day 1, the cell density was not significantly different between lamellar and dorsal shield blocks. From day 10 of cultivation, the cell density on the lamellar block was higher than that on the dorsal shield block. Furthermore, at day 15, proliferation of the cells cultured on the lamellar block was significantly higher than on the dorsal shield block (Fig. 3).

**Figure 4:**
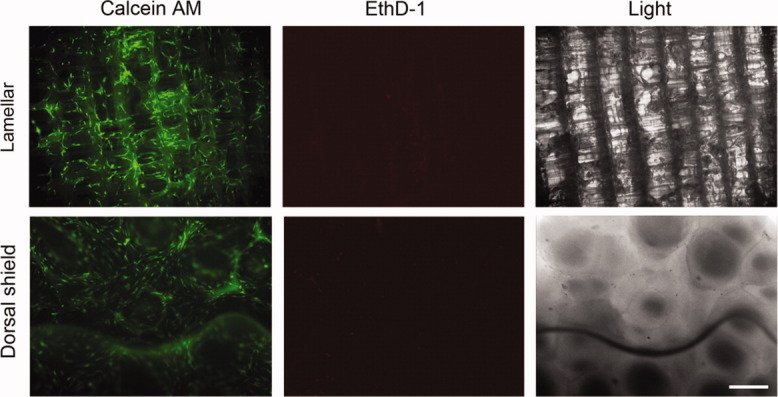
Cells were seeded on lamellar and dorsal shield blocks of cuttlefish bone and cultured in normal growth media. After 3 days of culture, the viability/cytotoxicity assay was performed. Calcein acetoxymethyl (AM) stained healthy cells green and ethidium homodimer-1 (EthD-1) stained the nuclei of dead cells red. Light images show the surface morphology of each block. Scale bar represents 50 μm. [Color figure can be viewed in the online issue, which is available at wileyonlinelibrary.com.]

To evaluate the cytotoxic effects of natural cuttlefish bone, cells were seeded and cultured on lamellar and dorsal shield blocks. After 3 days of cultivation in normal growth media, live/dead staining images were taken. The results showed that hMSCs were viable and very few dead cells were detected on both lamellar and dorsal shield blocks from cuttlefish bone. In addition, cells cultured on these blocks maintained a fibroblast-like morphology during cultivation (Fig. 4). These results indicated noncytotoxicity during hMSCs cultivation on natural cuttlefish bone.

**Figure 5:**
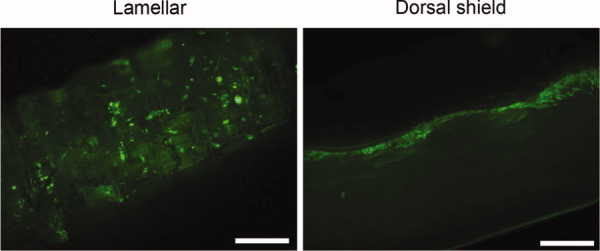
Fluorescence micrographs of stained cells seeded on lamellar and dorsal shield block sections after 3 days of culture. The sections were stained with calcein acetoxymethyl (AM). Cross sections of these blocks show that the cells were distributed inside the porous space of the lamellar block, whereas they only grew on the surface of the dorsal shield block. Scale bar represents 50 μm. [Color figure can be viewed in the online issue, which is available at wileyonlinelibrary.com.]

To assess the distribution of hMSCs throughout the blocks, the blocks were sectioned and stained with Calcein AM. The sections used were from the central part of the blocks and images were taken by using fluorescence microscopy. The fluorescence images clearly demonstrated a difference in cell distribution between lamellar and dorsal shield blocks. The seeded cells proliferated in a multilayer fashion and most of the cells were observed on the surface of the dorsal shield block, whereas on the lamellar block, the cells proliferated inside the pore channels after a period of 10 days (Fig. 5).

**Figure 6:**
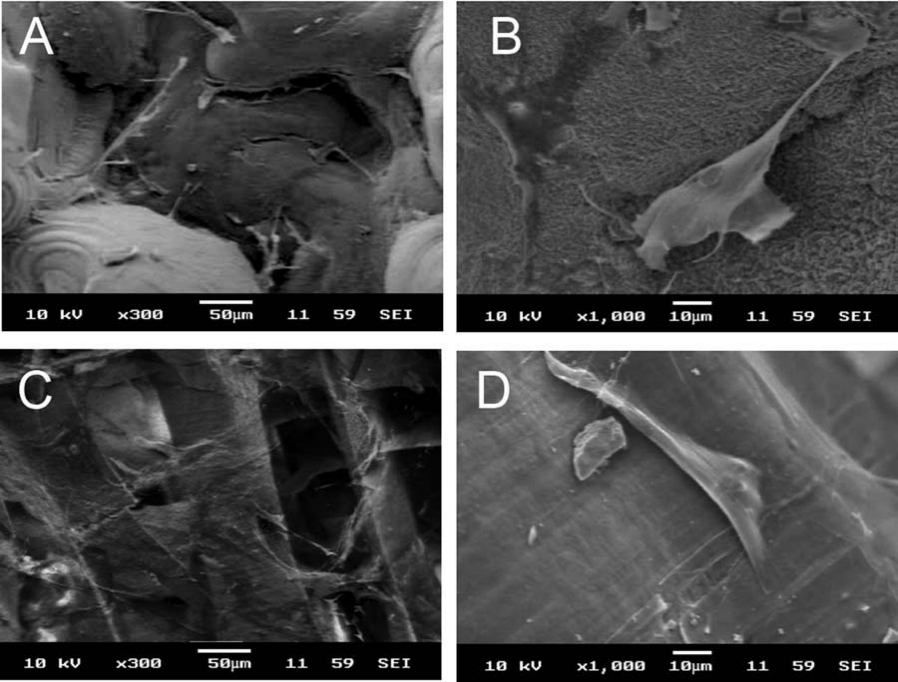
Human mesenchymal stem cells attached to the dorsal shield block (A and B) and lamellar block (C and D) of natural cuttlefish bone after 3 days of incubation (×300 and ×1000, respectively).

Cellular morphology and cell interaction of hMSCs seeded on lamellar and dorsal shield blocks were visualized by using SEM after 3 days of culture. On both the dorsal shield block and lamellar block, hMSCs grew following the surface morphology and maintained their fibroblast-like shape [Fig. 6(B,D)]. Furthermore, they formed bridges between the surface and spaces.

**Figure 7:**
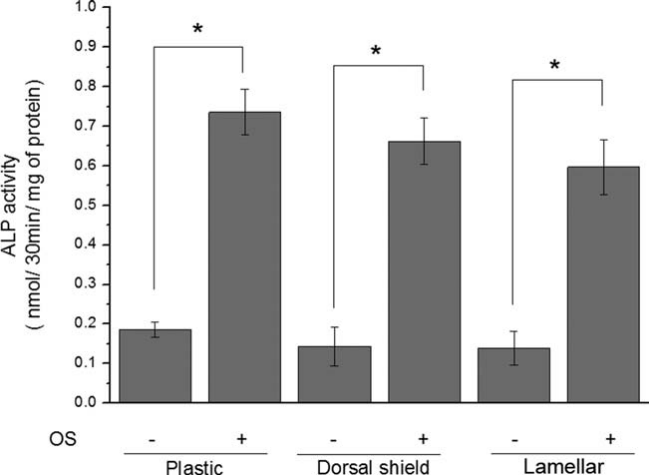
Alkaline phosphatase (ALP) activity of human mesenchymal stem cells (hMSCs) cultured on dorsal shield and lamellar blocks of natural cuttlefish bone. Cells were cultured for 5 days in the presence or absence of an osteogenic stimulant (OS). ALP activity significantly increased in hMSCs cultured on both dorsal shield and lamellar blocks. Values are expressed as the mean ± SD. Significance was determined at *p < 0.05 when compared with non-OS-treated cells.

**Figure 8:**
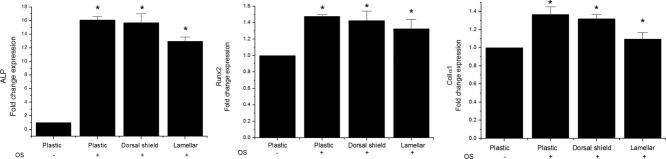
Osteogenic differentiation of human mesenchymal stem cells (hMSCs) cultured on plastic culture dish, dorsal shield block, and lamellar block. Cells were cultured for 5 days in the presence of an osteogenic stimulant (OS). Real-time polymerase chain reaction analysis for the expression of the osteoblast markers alkaline phosphatase (ALP), runt-related transcription factor 2 (Runx2), and collagen type I α 1 (ColIα1) showed a significant increase in hMSCs cultured on dorsal shield and lamellar blocks. Values are expressed as the mean ± SD. Significance was determined at *p < 0.05 when compared with non-OS-treated cells.

To determine whether hMSCs differentiated into osteoblasts during OS treatment and when cultured on natural cuttlefish bone, ALP activity was measured and qRT-PCRs were performed. The ALP activity of hMSCs was quantified after 5 days of culture (Fig. 7). The ALP values were normalized to the total protein content and expressed as nmol/mg protein per 30 min. When treated with OS, ALP activity increased in hMSCs cultured on both lamellar and dorsal shield blocks similar to that of cells cultured in plastic dishes (Fig. 7). Furthermore, ALP activity of hMSCs cultured on dorsal shield blocks was slightly higher than that on lamellar blocks. Similar results were observed for the mRNA expression level of ALP (Fig. 8). In addition, the expression of Runx2 and ColIα1 was significantly increased by OS treatment in cells cultured on both dorsal shield and lamellar blocks (Fig. 8).

## DISCUSSION

The development of porous materials, used as scaffolds for cell transplantation therapy, is of particular interest in tissue engineering. The biological responses of a material require the use of cell cultures adapted to tissue engineering. MSCs are multipotent stem cells that can differentiate into osteoblasts. Therefore, the behavior of MSCs in the presence of a scaffold material is an important indicator in the evaluation of the material's biocompatibility. The aim of this study was to evaluate natural cuttlefish bone as potential candidate for tissue-engineered bones. Natural cuttlefish bone is composed of calcium carbonate and contains small amounts of heavy metals and other organic components. These ancillary components have the potential to cause adverse reactions *in vivo*, such as an immune response. In this study, we used natural cuttlefish bone prepared with minimal processing and focused on ensuring that, by providing an appropriate environment, hMSCs attach, proliferate, and differentiate into osteoblasts.

First, we separated the cuttlefish bone into two structural parts (dorsal shield and lamellae) and confirmed the surface morphology of the lamellar and dorsal shield parts of natural cuttlefish bone. Birchall et al. have reported that cuttlefish bone (*Sepia officinalis L.*) consists of two regions: a thick external wall and an internal matrix. The dorsal shield is a hard cover that overlays the lamellar matrix. This layer has a nonporous structure. The lamellae are separated by pillars and form chambers, that is, areas with spaces between 200 and 600 μm.^14^ These microstructural properties of cuttlefish bone were confirmed in dorsal shield and lamellar blocks. Although we used cuttlefish bone of *Sepia esculenta* obtained from the Korean Sea, the lamellar pore size and structural patterns were not significantly different from previously observed results.^8^

The pore size is an important parameter for fluid flow in the scaffold, cell attachment, growth, and differentiation.^15^, ^16^ In an animal model, positive results seem to be achieved when scaffolds with a pore size in the range from 150 to 1000 μm are used.^17^ Comparison of cell proliferation at 15 days revealed that it was higher in cells cultured on the lamellar block than on the dorsal shield block (Fig. 3). Furthermore, cultured cells were distributed in the lamellar inner structure, but grew as a layer on the surface of the dorsal shield block. Therefore, these results show that compared with growth on the dorsal shield block, the three-dimensional surface area of lamellae provides additional support for cell growth.

Cell culture level tests are a starting point for establishing whether materials are noncytotoxic, and the tetrazolium-based colorimetric MTS assay is a quantitative method for assessing the biological response of cells to a material, whereas the direct contact test permits qualitative assessment of the cell's response after culture. According to the report by Cho et al., cuttlefish bone (*Sepia esculenta*) contains small amounts of heavy metals such as mercury (0.05 ppm), copper (0.52 ppm), zinc (2.42 ppm), lead (0.39 ppm), and cadmium (0.07 ppm).^18^ Therefore, we determined whether these heavy metals could affect cell proliferation and viability. Natural cuttlefish bone was used without any prior specific chemical treatment. Cells were cultured on lamellar and dorsal shield blocks for different periods of time (Fig. 3). In addition, in the viability/cytotoxicity assay, most of the cells appeared healthy and viable (Fig. 4). These results revealed that the heavy metals of cuttlefish bones have no cytotoxic effects on cell culture.^18^

The SEM study of hMSCs seeded on cuttlefish bone showed that hMSCs attached to both the lamellar and dorsal shield surface of cuttlefish bone blocks (Fig. 6). These results indicate that natural cuttlefish bone material allows the attachment and growth of hMSCs. Moreover, hMSCs maintained their typical fibroblast-like shape. This shows that cuttlefish bone serves as a good material for cell growth.

ALP activity is one of the most commonly used markers of osteogenesis and reflects the proportion of osteogenic differentiation. ColIα1 is known to play an important role during osteoblast differentiation and cell adhesion.^19^, ^20^ In addition, Runx2 is an osteogenic master transcription factor that plays a key role in osteoblast differentiation.^21^ In this study, ALP, ColIα1, and Runx2 were used as indicative markers for osteoblast differentiation. The ALP levels were increased during culture of hMSCs on dorsal shield and lamellar blocks. Furthermore, the ALP activity was higher in cells seeded on the dorsal shield block compared with those seeded on the lamellar block. This may be due to the fact that compared with lamellar blocks, cells cultured on dorsal shield blocks reached confluence earlier, which resulted in earlier differentiation. Intercellular communication affects the expression of differentiation markers of osteoblastic cells.^22^ Therefore, a higher cell density on the surface of dorsal shield blocks results in a higher degree of cell–cell interaction, and thus a higher rate of differentiation. In addition, the expression of Runx2 and ColIα1 was significantly increased in hMSCs during culture on both lamellar and dorsal shield blocks. These findings suggest that natural cuttlefish bone may be useful as scaffold for osteoblast differentiation.

## CONCLUSIONS

In this study, we have demonstrated that natural cuttlefish bone supports the adhesion, proliferation, and differentiation of hMSCs. In particular, hMSCs grew only on the outer surface of dorsal shield blocks, whereas they penetrated deeper into the lamellar blocks. These findings suggest that the lamellar and dorsal shield parts of cuttlefish bone could be potentially used as bone substitute and as a barrier to inhibit fibroblast infiltration near the bone defect during bone regeneration, respectively. However, one of the major disadvantages of the lamellar matrix of cuttlefish bone as bone regenerative scaffold is its fragility. Therefore, further study will be needed to improve its mechanical strength and stability.
